# Editorial: The role of E2F transcription factors in cancer

**DOI:** 10.3389/fonc.2023.1355610

**Published:** 2024-01-04

**Authors:** Ainhoa Iglesias-Ara, Bart Westendorp

**Affiliations:** ^1^ Department of Genetics, Physical Anthropology and Animal Physiology, University of the Basque Country, Bilbao, Spain; ^2^ Department of Biomolecular Health Sciences, Division Cell Biology, Metabolism and Cancer, Faculty of Veterinary Medicine, Utrecht University, Utrecht, Netherlands; ^3^ Regenerative Medicine Center, University Medical Center Utrecht, Utrecht, Netherlands

**Keywords:** E2F, cancer, biomarker, co-regulators, lung cancer, breast cancer, colon cancer

E2F transcription factors are the master regulators of cell cycle dependent gene expression driven by the CDK-RB-E2F axis. According to the canonical model, mitogenic signals lead to the activation of CDK4/6-cyclin D complexes, which phosphorylate and inactivate the retinoblastoma tumor suppressor. RB phosphorylation releases E2F transcription factors, which promote the expression of their target genes. E2Fs are involved in the transcriptional regulation of a large set of genes necessary for cellular proliferation, including genes involved in cell cycle progression, DNA replication, nucleotide biosynthesis, checkpoint control, and DNA repair. Importantly, many tumors show aberrant E2F activity as a result of mutations that happen upstream in the pathway. For example, inactivation of Retinoblastoma or overexpression of cyclin D. Although E2F transcription factors themselves are not as commonly mutated or altered as these upstream regulators, E2F3 is considered an oncogenic driver. Amplification of the *E2F3* gene is, for example, commonly seen in advanced bladder cancer (1).

E2F activity has been classically linked with cell cycle control but E2F target genes are also involved in a plethora of cell functions beyond cell cycle control. Additionally, the evidence showing new co-repressors and co-activators that interact with E2F to modulate its transcriptional activity is expanding. In line with this idea, the present Research Topic includes recent contributions from researchers presenting novel E2F target genes and E2F interacting co-regulators involved in the control of DNA damage response, apoptosis, stemness, cell proliferation, and oxidative protein folding. These roles illustrate the complexity of E2F-mediated regulation. Interestingly, research on this subject reinforces the utility of using E2Fs and some of their targets as potential biomarkers for early diagnosis and anticancer treatment.

In their contribution to the Research Topic, Xu et al. outline the discovery of a new E2F1-interacting protein that is upregulated in colorectal cancer, RING Finger, and WD Repeat Domain 3 (RFWD3). They show that RFWD3, a phosphorylation substrate of ATM/ATR that can respond to DNA damage by positively regulating p53 stability, participates in the occurrence and development of colorectal cancer. Interestingly, they show that RFWD3 interacts with E2F1 to potentiate the expression of the anti-apoptotic mediator BIRC5, also known as Survivin. Additionally, Gao et al. define a role for E2F3 to potentiate stemness and mesenchymal phenotypes in colon cancer cells. They propose that E2F3-mediated STAT3 pathway regulation could be partly responsible for the observed effects, although the molecular mechanism remains elusive. Nevertheless, these data support the idea that E2F3 could be a useful biomarker for anticancer treatment in colon cancer.

Xu et al. identified a novel target of E2F1 with relevance in breast cancer, Kinesine Family Member 26A (KIF26A). They show that elevated KIF26A expression is significantly correlated with lymph node metastasis of breast cancer. Their data support that KIF26A could promote proliferation and cell cycle entry in breast cancer cells. Mechanistically, they show that E2F1 transcriptionally activates KIF26A expression, which inhibits the CDK inhibitor P21, thus permitting the activation of the CDK-RB-E2F pathway in a positive feedback loop in breast cancer cells.

Li et al. present a study on non-small cell lung cancer (NSCLC), highlighting Quiescin Q6 sulfhydryl oxidase 2 (QSOX2). This enzyme can be directly secreted into the extracellular space. It promotes the formation of disulfide bonds in proteins and plays a key role in protein folding and stability. They show that QSOX2 is a putative biomarker in NSCLC, yet the intracellular and extracellular expression of QSOX2 by tumor cells markedly decreases after anti-cancer therapy *in vitro* and *in vivo*. They show that QSOX2 silencing in NSCLC cell lines results in the inhibition of cancer cell proliferation, induction of apoptosis, and decreased expression of cell division-related genes (CENPF and NUSAP1) and Wnt pathway activators (PRRX2 and Nuc-b-catenin). Mechanistically, they show that QSOX2 is a direct transcriptional target of E2F1. Because QSOX2 is a secreted protein, it can be measured in serum. Hence, these data support that this newly identified E2F target gene could be a useful biomarker for anticancer treatment in NSCLC.

The molecular actions of E2F transcription factors in cancer are complex and dependent on tissue context or cancer type. Their target genes affect proliferation, but also stemness and metastatic capacity. The papers in this Research Topic contribute to a better understanding of this complexity by showcasing novel E2F-interacting proteins and E2F target genes. Further research is required to validate their potential clinical value in biomarker development.

## Author contributions

AI-A: Writing – original draft. BW: Writing – review & editing.
